# Supplementary material for: Shear Wave Elastography for measuring the elastic properties of the Psoas Major muscle: An intra- and inter-examiner reliability study
Source: PLoS One. 2025 Oct 9;20(10):e0330076. doi: 10.1371/journal.pone.0330076 (PMC12510488; doi:10.1371/journal.pone.0330076)
Supplement: S1 File — (PDF) [file pone.0330076.s001.pdf]

Shear wave speed Examiner 1 Trial 1	Shear wave speed Examiner 1 Trial 2
2.29	2.31
2.01	2.19
2.32	2.29
2.23	2.25
2.47	2.50
2.68	2.67
1.75	1.75
2.66	2.50
1.75	1.67
1.99	1.97
2.17	2.00
2.13	2.18
2.68	2.58
1.84	1.70
1.97	1.90
2.52	2.41
2.28	2.41
1.88	1.87
2.27	2.38
2.79	2.71
1.99	1.99
1.57	1.67
2.05	2.08
2.59	2.56
2.05	2.22
2.20	2.24
2.28	2.30
2.41	2.18
2.35	2.26
1.72	1.61
2.17	2.22
1.94	2.02
1.93	2.08
2.49	2.44
2.46	2.51
2.10	2.17
2.64	2.59
2.51	2.53
1.89	1.92
2.51	2.42
2.27	2.32
1.71	1.73
2.23	2.47

2.12	2.19
2.57	2.48
1.80	1.89
1.82	1.86
2.13	2.15
2.67	2.58
2.14	2.01
2.36	2.05
2.55	2.58
1.90	1.86
1.54	1.61
2.63	2.73
2.52	2.37
2.18	2.24
2.10	1.97
2.91	2.71
2.61	2.67
1.86	1.71
2.05	1.99
2.44	2.31
1.79	1.88
2.26	2.20
1.64	1.64
1.98	2.07
1.98	2.09
2.04	2.12
1.86	1.79
1.93	1.92
2.12	2.20
1.55	1.61
1.78	1.62
1.54	1.61
1.54	1.61
2.33	2.48
1.80	1.99
1.69	1.92
2.13	2.14
2.07	2.05
2.18	2.42
2.17	2.27
1.54	1.61
2.10	2.09
1.78	1.72
2.56	2.52

1.85	2.00
2.38	2.34
2.69	2.74
2.36	2.58
2.07	1.97
2.37	2.43
2.59	2.58
1.89	1.97
2.21	2.17
2.04	2.04
2.04	1.95
2.18	2.20
2.48	2.39
1.88	2.06
2.21	2.11
2.17	2.34

Young's Modulus Examiner 1 Trial 1	Young's Modulus Examiner 1 Trial 2
2.00	2.24
1.90	1.96
2.03	1.77
2.11	1.92
2.26	2.56
2.34	2.41
2.18	1.92
2.83	3.03
1.88	2.06
2.11	2.19
2.05	2.21
2.30	2.25
2.34	2.26
1.80	2.01
1.73	1.86
2.31	2.15
2.00	2.07
1.66	1.86
2.16	2.33
2.67	2.76
1.88	2.17
2.11	2.27
2.05	1.87
2.51	2.82
1.91	2.00
2.09	2.33
2.04	2.28
1.83	1.95
2.19	2.28
1.87	1.87
2.04	2.01
1.70	1.62
1.85	1.71
2.27	2.46
2.26	2.43
1.85	1.91
2.36	2.64
2.27	2.46
2.25	2.27
2.27	2.33
2.13	2.12
1.87	2.00
2.11	2.16

1.51	1.46
2.32	2.36
1.78	1.95
1.78	1.80
2.04	1.85
2.46	2.54
2.04	2.37
2.20	2.09
2.31	2.50
1.62	1.86
1.87	2.03
2.58	2.72
2.22	2.17
2.07	2.17
1.98	1.97
2.39	2.41
2.53	2.48
1.81	2.04
1.92	1.90
1.77	1.97
1.70	1.64
2.19	2.48
2.18	2.14
1.55	1.55
1.85	1.98
1.90	2.20
1.81	1.94
1.85	1.73
1.62	1.91
1.51	1.75
1.70	1.65
1.63	1.76
1.65	1.64
2.18	2.33
1.83	1.63
1.86	1.46
1.99	1.94
1.98	1.75
2.06	1.99
1.91	2.04
1.63	1.54
1.98	1.99
1.77	1.75
2.32	2.26

1.80	1.94
2.23	2.25
2.64	2.92
2.19	2.44
1.94	1.89
2.20	2.43
2.33	2.43
2.23	2.25
2.11	2.26
1.91	1.71
1.90	1.74
2.09	2.26
2.26	2.40
1.83	1.92
1.51	1.41
2.06	1.96

Shear wave speed Examiner 2 Trial 2	Shear wave speed Examiner 1 Trial 2
10.98	9.99
15.95	16.55
22.57	21.70
20.87	20.42
17.82	15.98
14.06	14.42
18.68	19.29
10.46	13.82
7.80	9.24
7.80	8.40
17.46	18.81
8.39	8.40
9.96	9.79
12.57	14.21
19.86	19.20
13.93	14.23
14.78	17.93
20.38	20.73
13.57	13.27
26.93	27.84
14.42	13.45
15.02	12.86
19.48	21.09
11.87	11.74
18.38	19.06
18.11	19.40
13.15	14.25
14.75	14.84
16.35	18.29
10.99	12.89
9.67	10.43
21.44	17.93
17.09	16.20
19.00	16.19
14.04	15.17
14.65	13.39
10.70	11.29
12.18	11.45
23.95	23.65
11.52	15.74
20.45	20.16
18.30	18.92
18.53	17.92

12.15	13.78
28.65	28.50
16.55	16.80
19.99	19.21
12.98	15.33
24.25	23.96
11.76	12.97
7.80	8.40
8.24	8.76
15.59	16.30
15.00	14.69
14.79	12.50
11.84	13.20
13.11	11.89
22.68	20.05
23.55	22.76
15.68	13.49
17.36	20.26
14.95	14.80
11.17	10.63
10.03	10.24
17.70	19.78
26.84	26.13
10.03	12.02
14.41	14.77
7.80	8.40
21.02	21.11
19.87	18.65
20.55	19.01
19.26	20.17
18.52	17.28
17.79	18.65
18.51	18.01
18.81	17.97
16.58	14.75
20.70	17.08
22.13	20.81
25.26	24.28
14.97	15.72
12.15	12.11
13.84	12.89
19.64	19.65
7.80	8.40
19.41	18.92

13.71	14.54
16.46	15.60
14.63	13.93
7.84	9.11
7.80	8.40
14.81	15.43
20.61	20.16
16.00	16.59
19.57	17.96
21.15	21.26
16.09	18.15
9.33	10.10
7.80	8.40
9.08	10.25
10.45	13.86
20.64	20.42

Young's Modulus Examiner 2 Trial 2	Young's Modulus Examiner 2 Trial 2
15.98	10.72
17.33	16.65
21.78	23.56
18.40	21.88
17.68	17.79
10.98	13.66
18.25	19.06
10.45	10.43
8.60	6.64
11.72	9.22
16.56	17.67
8.60	8.90
10.49	13.33
9.53	12.35
17.99	19.97
11.62	13.44
12.17	14.56
15.08	20.18
12.44	13.12
23.50	31.69
12.75	13.77
17.12	15.93
16.45	19.77
14.50	11.86
16.26	18.20
12.91	18.08
10.89	12.97
11.24	14.49
14.66	16.95
10.32	10.82
10.92	9.10
17.62	22.30
14.63	17.58
13.81	19.15
15.14	13.47
16.84	14.48
10.25	10.66
8.72	12.29
21.73	25.70
10.15	11.33
28.23	34.30
14.98	18.11
20.38	19.03

13.98	12.12
18.68	20.38
10.93	17.14
18.87	20.10
11.19	12.51
25.73	26.15
8.60	11.70
15.86	16.43
9.55	8.56
18.85	16.34
11.09	15.33
14.58	15.12
15.92	11.80
9.27	12.56
18.26	24.16
18.78	24.95
8.60	6.60
17.31	17.60
16.64	15.25
14.03	10.86
8.60	6.60
10.76	17.75
26.23	29.59
14.34	9.31
14.98	13.73
8.60	6.93
23.26	21.91
22.02	19.98
17.61	20.45
18.95	19.72
16.01	18.40
16.47	17.77
14.07	18.27
19.63	19.13
11.94	17.57
23.33	21.82
18.16	22.93
21.01	27.71
17.11	15.29
8.60	11.89
17.11	13.36
15.11	19.91
10.40	7.92
20.24	19.73

8.60	6.60
12.42	17.14
12.08	13.98
8.60	8.13
17.42	10.33
16.30	15.17
20.38	20.81
14.56	16.84
17.68	19.79
24.57	22.18
15.14	16.94
8.60	9.02
15.64	9.88
8.81	8.93
9.19	6.60
16.48	20.82
